# Distribution of Sequencing Coverage Gaps in Exomes and Genomes: Potential Implications for Diagnostic Accuracy in Neurodevelopmental Disorder Genes

**DOI:** 10.3390/genes17030269

**Published:** 2026-02-26

**Authors:** Emanuela Iovino, Claudia De Masi, Anna Ballestrazzi, Alessandro Mattiaccio, Federica Isidori, Marco Seri, Tommaso Pippucci

**Affiliations:** 1 IRCCS Azienda Ospedaliero-Universitaria di Bologna, 40138 Bologna, Italyalessandro.mattiaccio@aosp.bo.it (A.M.);; 2 Department of Pharmacy and Biotechnologies, University of Bologna, 40138 Bologna, Italy; anna.ballestrazzi@studio.unibo.it

**Keywords:** exome sequencing, whole genome sequencing, coverage gap, coverage quality control, clinical utility

## Abstract

Background: Exome (ES) and genome sequencing (GS) are powerful tools for diagnosing neurodevelopmental disorders (NDDs), yet sequencing coverage failures can leave clinically relevant variants undetected. Analyzing the distribution of coverage gaps across sequencing approaches and batches is therefore informative for diagnostic accuracy. Methods: We analyzed sequencing data from 43 NDD patients across four ES runs, including 14 individuals sequenced by both ES (Twist Human-Core-Exome-v1.3) and GS. Low-coverage regions (LCRs) were defined as target intervals with mean depth <20 x, and z-scores < −1.96 were used to identify batch-specific systematic LCRs. LCRs were clinically annotated using OMIM and SysNDD databases. Results: LCR patterns were highly consistent within each ES batch but were characterized by extreme variability between batches. Higher global mean coverage increased intra-batch consistency, but batches sequenced at a commonly accepted yield in clinical sequencing (>100 x mean coverage) showed thousands of batch-specific LCRs. LCR patterns substantially diverged between ES and GS, displaying preferential impact on different genes. Although a restricted group of genes accumulates LCRs disproportionately, most LCRs are broadly dispersed throughout the genome. LCRs were not systematically associated with features such as GC content and genomic location (e.g., exon 1). Interestingly, LCRs affected OMIM/SysNDD genes and occasionally overlapped ClinVar pathogenic variants, indicating potential impact on diagnostic sensitivity. Conclusion: The global distribution of coverage gaps appears strongly influenced by batch-specific effects, making the occurrence of LCRs partly unpredictable even within clinically relevant gene sets. These findings support systematic assessment of LCRs as a component of quality evaluation in diagnostic sequencing workflows.

## 1. Introduction

Over the last couple of decades, the progressive adoption of exome sequencing (ES) and genome sequencing (GS) in the clinical practice of rare genetic diseases (RGDs) has unquestionably improved diagnostic rates and expedited routine analysis of hundreds to thousands of genes in highly genetically heterogeneous conditions. Despite their transformative effects, both ES and GS are prone to technical biases that impact coverage depth, uniformity, and ultimately variant detection sensitivity [1]. Genomic features such as extreme GC content and segmental duplications, as well as processing-related factors including library preparation, amplification, and downstream computational elaborations, can affect Depth of Coverage (DoC), defined as the number of sequencing reads mapping to a given nucleotide position [1,2]. Furthermore, hybridization-based ES capture introduces additional variability, as uneven probe performance and differential enrichment efficiency often produce less uniform coverage than that typically achieved with GS [3]. The extent and recurrence of low-coverage regions (LCRs) remain poorly investigated; nonetheless, LCRs may hide clinically relevant variants, therefore influencing the rate of false negative clinical findings in ES- and GS-based diagnostics [4].

Pathogenic variants that are technically challenging for next-generation sequencing (NGS) are widespread across many genes in a large and heterogeneous patient cohort [5]. In some settings, pathogenic variants that are readily detectable by targeted NGS panels exhibited low or no coverage in some ES workflows, indicating that variability in exome depth can substantially reduce detection sensitivity [5]. These observations underscore that, taken alone, global DoC metrics may lead to overlooking the failures of ES and GS in effectively representing every region they are supposed to cover.

Neurodevelopmental disorders (NDDs), characterized by extreme genetic heterogeneity, greatly benefited from the introduction of ES and GS as first-tier diagnostic tests [6,7,8,9,10,11,12,13].

A standardized characterization of LCRs is therefore crucial for optimizing diagnostic pipelines, especially for vastly genetically heterogeneous conditions such as NDDs, and for improving quality control across sequencing platforms [14]. This approach can help in systematically identifying exons and genes impacted by LCRs, highlighting specific regions prone to possible false negative findings. To support this type of assessment, tools such as unCOVERApp (v.2.1) provide an interactive visualization and functional annotation of LCRs within target regions and help integrate coverage information into clinical interpretation workflow [4].

In this study, we investigated whether LCRs are predictable and specific to sequencing approaches, such as ES versus GS, or instead reflect variability from sample to sample or batch to batch that cannot be inferred from global coverage metrics alone. We conducted a comprehensive comparison of ES and GS data derived from families affected by NDDs, including samples sequenced using the Twist Human Core Exome v1.3 capture system and systematically identified LCRs. These regions were annotated using curated databases of NDD-associated genes (OMIM, SysNDD) and a recently updated version of unCOVERApp.

Collectively, we carried out a composite analysis to clarify the origin, distribution, and predictability of LCRs in clinical ES/GS workflows and to provide practical tools for incorporating coverage gaps into the interpretation of negative genomic test results in NDD diagnostics.

## 2. Materials and Methods

### 2.1. Sequencing Data Selection and Generation of DoC Metrics

We randomly selected 43 individual ES data, separated into groups of samples each from 4 sequencing runs carried out as part of the Rare Genetic Diseases (RGDs) ES diagnostic program at U.O.C. Medical Genetics, IRCCS Azienda Ospedaliero-Universitaria di Bologna. In the framework of this program, genomic DNA samples obtained from peripheral whole blood of RGD patients were sent for ES at Personal Genomics (Verona, Italy). ES libraries were prepared with Twist Human Core Exome Kit v.1.3 (TWIST Bioscience HQ, South San Francisco, CA, USA) and sequenced on Illumina NovaSeq 6000 sequencing system as 150 bp paired-end reads. To conduct intra-batch and inter-batch comparisons of DoC in ES data, we created four batches of the same sample size, exemplifying the results of independent ES runs (1–4 in Table 1), ranging from a per-batch mean DoC of 117 x to 229 x (Table 1). A subset of 14 ES data with a mean sequencing DoC of 225.5 x was selected to compare LCRs in ES and GS, leveraging the GS performed for the same patients. Indeed, the DNA samples belonging to the same patients underwent GS (per-batch mean DoC of 64.4 x) in the context of a research project aimed at GS-based resequencing of undiagnosed patients with neurodevelopmental disorders (NDDs) (per-batch mean sequencing DoC of 64.4 x). Genomic DNA was prepared with the Illumina TruSeq Nano DNA Library Prep kit (Illumina, CA, USA) and sequenced on the NovaSeq 6000 sequencing system as 150 bp paired-end reads.

ES reads were aligned to the GRCh38 reference genome with BWA-MEM (v0.7.17) [15,16], followed by duplicate marking and base quality score recalibration according to GATK Best Practices (GATK v4.0) [17]. GS data were processed using the Illumina DRAGEN Germline Pipeline (v4.0.3) via BaseSpace (DRAGEN V.4.0.3, Illumina, San Diego, CA, USA). DoC was calculated with Mosdepth (v0.3.0) [18] using coding DNA sequence (CDS) intervals derived from GENCODE v35 annotations as target regions. For each CDS interval, Mosdepth computed the mean DoC, and all downstream analyses for ES and GS were based on these per-interval mean DoC values, using the same CDS target set for both. Default Mosdepth settings were applied, and no additional quality filters were set, thereby using all aligned reads for the DoC estimates.

### 2.2. Definition and Analysis of Low Coverage Regions

Low coverage regions (LCRs) were defined at the level of CDS intervals, using the mean DoC computed by Mosdepth for each interval. An interval was classified as an LCR if its mean DoC was below 20 x in at least one of the 14 ES and GS data included in the ES-GS comparison. To ensure strict comparability between the two, this analysis was restricted to CDS intervals intersecting the ES target design. A 20 x DoC was chosen as it is the generally recognized threshold for adequate identification of heterozygous and homozygous germline variation in NGS data. A schematic overview of the analytical workflow, from sequencing and alignment to coverage calculation and LCRs annotation, is shown in Supplementary Figure S1.

### 2.3. Intra-Sample Comparison of ES and GS

For each individual sample sequenced by both ES and GS, we quantified the similarity between low-coverage profiles using the Jaccard similarity coefficient, defined as the ratio between the number of genomic intervals classified as LCRs in both ES and GS and the total number of intervals classified as LCRs in at least one approach. In this framework, a Jaccard value of 1 denotes complete concordance, with ES and GS identifying exactly the same set of intervals as LCRs, whereas values tending to 0 indicate that the two assays designate increasingly non-overlapping sets of LCRs. To assess whether ES and GS exhibited concordance in their low-coverage patterns, we tested the distribution of per-sample Jaccard coefficients being significantly <1 using a one-sample Wilcoxon signed-rank test. While the Jaccard similarity coefficient quantifies, for each individual, the direct overlap between ES and GS LCRs, principal component analysis (PCA) was used to investigate global patterns in the full low-coverage profile of each sample and sequencing approach, using binary representations of LCRs at each interval. To this end, we constructed a binary matrix with genomic intervals as rows and samples as columns, assigning a value of 1 when an interval was classified as an LCR in a given sample and 0 otherwise. This multivariate representation enabled ES and GS samples to be jointly projected into a common principal component space, where similarities and differences in their global low-coverage profiles could be assessed.

### 2.4. GC Content Analysis

GC content for each LCR at the gene level was calculated using bcftools (v1.9). GC content distributions were compared among ES-only, GS-only, and shared LCRs using a Kruskal–Wallis test. Pairwise comparisons between LCR categories were performed using Dunn’s post hoc test with Benjamini–Hochberg correction for multiple testing.

### 2.5. z-Score Standardization and Intra-Batch Analysis

To identify systematic LCRs (sLCRs), we first isolated the LCRs of batches 1–4. In each individual ES data, we standardized the maximum DoC, capping at 20 x any LCR single nucleotide position originally ≥20 x, and calculated x as the LCR mean DoC. For each batch, we standardized coverage by calculating a z-score for each interval as

z-score = (mean depth − batch mean)/batch SD

where the batch mean and SD were computed across all samples in each batch. Intervals with z-score < −1.96 (i.e., substantially below the batch average) were identified as batch-specific LCRs. After defining batch-specific LCRs, we assessed their reproducibility within each batch. For intra-batch assessment, each interval was classified as “consistent” when all samples within a batch showed the same status (all LCR or all adequate coverage). The proportion of consistent intervals reflects whether coverage variability tends to affect all the samples in a batch uniformly or only partially. For inter-batch comparison, we identified intervals that were consistently low-covered (sLCRs with Z < −1.96) in each batch separately. Their intersections distinguished genomic regions with recurrent LCRs across multiple batches from those restricted to single batches.

### 2.6. Genomic and Functional Annotation of LCRs

LCRs were annotated using Gencode.v49 to determine their genomic context, including co-localization within protein-coding genes and coding exons. Using GENCODE v49 annotations, we aggregated LCRs by gene and evaluated two complementary metrics. At the gene level, we defined binary indicators for the presence of LCRs in coding exon 1 and in other coding exons to assess whether the involvement of exon 1 differed from that of other coding exons at the gene level. At the base-pair level, we quantified low-coverage burden as the fraction of CDS bases overlapping LCRs in each region, enabling comparisons normalized by coding sequence length. LCRs were classified as ES-only, GS-only, or shared (detected in both ES and GS). Moreover, we evaluated whether LCRs concentrate in a subset of genes. To visualize gene-level LCR burden, accounting for differences in coding sequence length, LCR counts were normalized by CDS length. For each gene, the number of ES-only, GS-only, and shared LCRs was divided by the total coding sequence length expressed in kilobases (kb), yielding length-normalized LCR rates (LCRs per kb of CDS) which directly capture the density of LCRs along the coding sequence and are less sensitive to differences in exon number and size across genes. Functional annotation was performed using the updated unocevarppLib (https://github.com/emanuela04/uncoverappLib/tree/versione_2.1, accessed on 1 January 2026), which integrates OMIM morbid genes (February 2024), variants reported in ClinVar as pathogenic or likely pathogenic, in silico predictors such as CADD scores, together with the SysNDD (2023) neurodevelopmental disorder gene list, restricting NDD-associated genes to those with definitive or moderate evidence of disease association. This framework enabled the identification of clinically relevant genes affected by LCRs and sites where deleterious variants may be missed because of falling into an LCR.

### 2.7. Statistical Analysis

All statistical analyses were performed in R v4.5. For ES-GS comparison, differences between ES and GS LCRs were assessed using a Kruskal–Wallis test followed by Dunn’s post hoc comparisons with Benjamini–Hochberg correction. For batch variability analyses, differences in LCRs across batches were tested by comparing z-score distributions using Kruskal–Wallis and pairwise Wilcoxon rank-sum tests with Benjamini–Hochberg correction. PCA and hierarchical clustering heatmaps of binary LCR matrices were used to assess multivariate sample clustering and visualize batch-dependent structure.

## 3. Results

### 3.1. Intra-Sample Comparisons Reveal Distinct LCRs Between ES and GS

**Figure 1:**
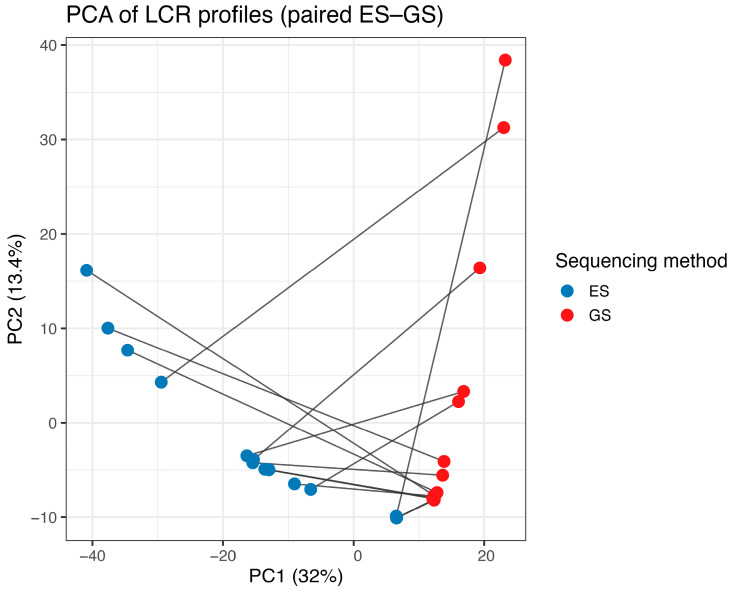
PCA of LCRs derived from ES and GS. Each point represents the LCR profile of one sample for either platform, with lines linking paired ES–GS measurements from the same individual. ES (blue) and GS (red) form clearly separated clusters, and paired profiles do not cluster together, indicating that low-coverage signatures are largely platform-specific rather than sample-intrinsic.

In all the samples analyzed by both ES and GS, LCRs represented a very small minority of the target space. LCR proportions were similar between ES and GS despite the highly different mean DoC of the two settings. In fact, LCRs ranged from 0.08% to 2.93% (median value: 0.72%) in ES and from 0.12% to 1.75% (median value: 0.18%) in GS. Nonetheless, when considering unique genomic intervals, only 943 LCRs were shared between ES and GS, while 7249 were specific to ES and 4861 to GS. The per-sample amount of ES-only LCRs was significantly higher than GS-only LCRs (paired Wilcoxon *p* = 0.018). The two measures were not correlated (Spearman ρ = 0.08), showing no homogeneous trend in the relative abundance of LCRs between ES and GS (Figure 1). Variance in LCRs was comparable between ES and GS (Levene’s test *p* = 0.31), confirming that the two sequencing methods exhibited similar levels of sample-to-sample variability. Moreover, LCR overlap between ES and GS performed on the same sample was systematically low (median Jaccard = 0.04; Wilcoxon vs. J = 1, *p* = 6.1 × 10^−5^), indicating that only a small fraction of intervals classified as LCRs in one assay were also low-covered in the other. PCA of the binary LCR matrix was consistent with these findings. ES and GS clustered by sequencing method and not due to originating from the same sample or patient, suggesting approach-specific patterns in LCR profiles (Figure 1). To evaluate the robustness of the ES-GS comparison to the depth threshold used to define LCRs, we repeated the analyses using alternative cut-offs of 10 x and 30 x. These thresholds changed the absolute number of intervals classified as LCRs but did not alter the qualitative patterns, including the limited overlap between ES and GS LCRs (Figure S2, Table S1).

### 3.2. GC Content Distinguishes ES-Only from GS-Only LCRs

**Figure 2:**
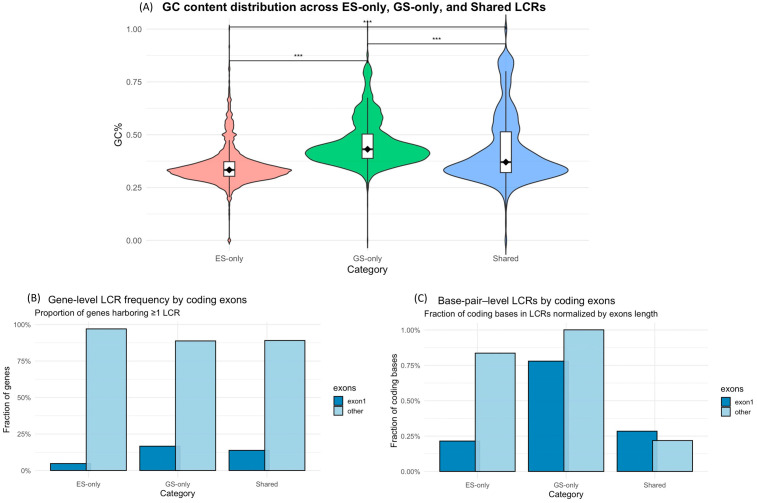
(**A**) GC-content distribution in ES-only, GS-only, and shared LCRs. GS-only intervals show significantly higher GC content compared with ES-only and shared LCRs. Statistical significance is indicated by asterisks (*** *p* < 0.001) (**B**) Gene-level frequency of LCR occurrence across coding exons. The plot shows, for each LCR category (ES-only, GS-only, Shared), the proportion of genes with at least one LCR in coding exons. Genes are further stratified according to whether LCRs overlap exon 1 or downstream coding exons, highlighting how often the first coding exon is affected compared with internal exons. (**C**) Base-pair level distribution of LCRs across coding exons. For each LCR category, the plot reports the percentage of coding bases (GENCODE target regions) overlapping LCRs, separately for exon 1 and for internal coding exons. This base-pair normalized view allows comparison across exons of different lengths and positions, disentangling effects driven by the larger size of exon 1 versus other exons.

GC content differed significantly among the three sets of LCRs (Kruskal–Wallis *p* < 2 × 10^−16^), and pairwise Dunn tests with Benjamini–Hochberg correction confirmed significant differences for all comparisons. ES-only LCRs showed the lowest median GC (0.333), GS-only LCRs had substantially higher GC (0.431), closer to the genomic average and reflecting the more uniform GS global coverage. Shared LCRs displayed an intermediate median (0.370) and a broader range, indicating that LCRs for both methods tend towards both GC extremes (Figure 2A).

### 3.3. LCRs Are Broadly Distributed Across Genes

Genes were more likely to contain an LCR in any other exon than in exon 1 (Figure 2B), consistent with greater cumulative length of non-1 exons. Even after normalization for exon length, exon 1 was not preferentially affected by LCRs, as the rate of non-1 exons exceeded that of exon 1 in both ES-only and GS-only. In shared LCRs, exon 1 was represented only slightly and non-significantly more than non-1 exons (Figure 2C). LCRs were observed in 4427 genes. Absolute LCR counts clearly reflected CDS size (e.g., TTN). After normalizing by CDS length, the highest LCR count percentile revealed no predominant origin of LCRs among ES-only, GS-only, and shared regions (Table S2), and normalized LCR rates for OMIM and SysNDD genes are displayed in Figure S4 to illustrate this distribution in clinically relevant loci. Taking as an example the top-ranking 100 genes, these accounted for ~18% of LCRs, a proportion considerably higher than expected under a uniform distribution (~2.3%). Nonetheless, the remaining 82% were broadly distributed, showing that LCRs are not confined to a small set of problematic genes.

### 3.4. LCRs Affect Clinically Relevant Genes and Overlap with Known Pathogenic Variants

**Figure 3:**
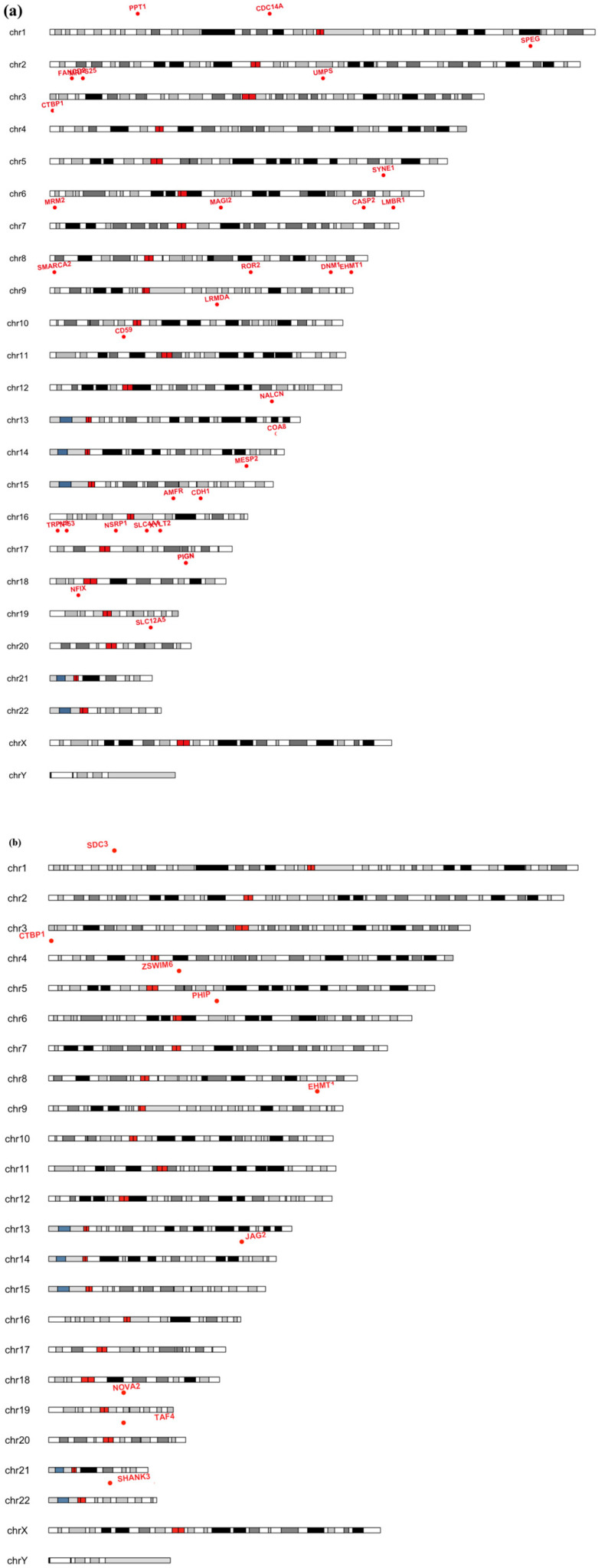
Genomic distribution of recurrent LCRs in ES and GS. Ideograms show chromosomes with genes affected by recurrent LCRs in exome sequencing (**a**) and genome sequencing (**b**). Red labels indicate OMIM genes. The limited overlap between ES and GS illustrates that LCRs affect distinct gene sets depending on the sequencing method, while involving clinically relevant loci in both datasets.

To assess the potential clinical relevance of LCRs in NDD diagnostics, we first quantified the global burden of LCRs across all NDD samples using the LCR definition and gene annotations described in the Methods. Across the NDD cohort, 100% of samples harbored at least one LCR overlapping an OMIM gene. Considering the number of LCRs per individual in NDD-associated genes, samples exhibited a median of 140 LCRs in the ES dataset and 20 LCRs in the GS dataset (Table S3). In addition, 27% of SysNDD genes in ES and 13% in GS were affected by at least one LCR in at least one sample, showing that coverage gaps in disease-associated genes occur with a frequency that may have practical implications for diagnostic interpretation. Functional annotation using the updated uncoverappLib revealed that LCRs may have direct implications for clinical diagnostics. In ES, recurrent LCRs present across all samples are mapped to 22 OMIM genes (Figure 3a), of which eight are associated with NDD according to the SysNDD database: CTBP1, COA8, MRPS25, PIGN, PPT1, SLC12A5, SLC35A3, and SMARCA2. Importantly, some LCRs directly overlapped known pathogenic variants reported in ClinVar. As an example, PIGN (OMIM: 606097) had recurrent LCRs at a locus harboring multiple pathogenic variants (e.g., NM_176787.5:c.755A>T; p.Asp252Val) associated with NDD, which may be missed due to insufficient DoC (Figure S5). In the GS dataset specifically, seven OMIM genes were affected by recurrent LCRs (Figure 3b), of which five are associated with NDD: CTBP1, JAG2, PHIP, SHANK3, and TAF4. Notably, SHANK3, a well-established NDD gene, harbored an LCR encompassing a missense variant of uncertain significance with high pathogenicity scores (NM_001372044.2(SHANK3):c.1598C>T (p.Pro533Leu), ClinVar: RCV000585233).

### 3.5. Batch Effects in ES-Only LCRs

**Figure 4:**
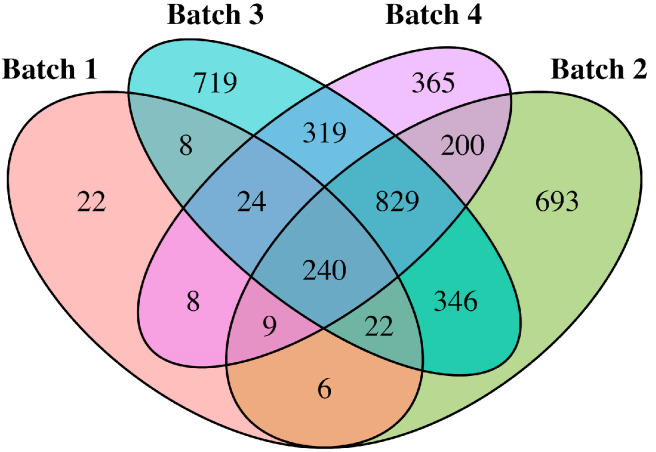
Venn diagram showing the overlap of LCRs across the four sequencing batches. Each ellipse represents one batch, and numbers indicate the count of LCRs unique to each batch as well as those shared among different combinations of batches. The diagram highlights both batch-specific LCRs and regions recurrently detected across multiple batches.

Only 240 LCRs were present across the four ES batches (Batches 1–4), outnumbered by batch-specific LCRs (Figure 4).

Investigating the consistency of LCRs within each batch, we observed 98%, 81.5%, 80% and 84.5% consistency for batches 1–4, respectively. This indicates that the vast majority of LCRs are systematic within the sequencing batch and suggests that a higher average DoC may minimize sample-to-sample variability, as supported by the higher consistency of batch 1 compared to the others (Table 2, Figure S6). We also explored different z-score thresholds for defining systematic LCRs within ES batches (z ≤ −1.64, −1.96, and −2.58). While more stringent cut-offs reduced the number of intervals labelled as systematic LCRs, the evidence for clear batch-dependent LCR profiles and their reproducibility within batches remained essentially unchanged (Figure S7, Table S4).

Multivariate analysis (PCA; Figure S8) confirmed batch-dependent structure, with Batch 1 forming a compact group and Batches 2–4 showing partially overlapping but still distinguishable patterns. Samples tended to cluster according to batch of origin, indicating systematic rather than random variation with highly significant differences in LCR-associated z-scores across batches (Kruskal–Wallis *p* < 2.2 × 10^−16^) and multiple significant pairwise contrasts identified by Wilcoxon tests. Overall, these results demonstrate that LCRs capture consistent batch-dependent effects and that differences between batches are systematic.

## 4. Discussion

With the pervasive use of ES and now increasingly GS in the RGD clinical setting, a persistent challenge is understanding how uneven coverage affects the sensitivity of these assays at the granularity level of single genes or exons [19]. In this study, we first provided a thorough and composite analysis of LCR variability within and between sequencing batches. We found that LCRs are not dominated by a small set of stereotypically challenging regions, such as exon 1, but likely reflect largely batch-specific library issues. While batch effects in sequencing data have been documented in terms of variant quality and systematic biases across sequencing centers, their impact on the genomic distribution of LCRs and their implications for variant calling in disease genes have not been systematically characterized [20,21]. We showed that while intra-batch LCR consistency was high, only a small subset of LCRs was shared by four independent batches using the same Twist Human Core Exome version (1.3) enrichment capture system and Illumina sequencing. Intra-batch consistency was particularly pronounced in the batch with the highest mean DoC, suggesting that higher global coverage yield limits the randomness of LCR distribution. Accordingly, the three batches with mean depths closer to those typically used in routine diagnostics (117 x, 117 x, and 126 x) still exhibited a greater amount of LCRs with markedly distinct LCR structure. Importantly, shifting from ES to GS does not eliminate LCRs and the unpredictability of their distribution pattern. This observation warns about the notion of “whole” genome sequencing. In fact, GS is commonly seen as an ultimate approach not only to capture extra-coding regions but also to better characterize coding sequences missed by ES. Our findings suggest that one of the reasons why negative GS results are not necessarily true is the occurrence of LCRs. Most importantly, GS performed after inconclusive ES may be characterized by a largely non-ES-overlapping LCR set. Therefore, while GS adequately covers most of the regions where ES fails, it introduces original LCRs, suggesting that using GS as a first-tier test would still not provide actual “whole” genome coverage, even limited to coding exons. Clinically relevant NDD genes harbored recurrent LCRs exclusively seen either in ES or GS, as, respectively, PIGN and SHANK3. Although in the clinical setting it may be prohibitive to cover all LCRs with subsequent analyses using orthogonal techniques such as Sanger sequencing, LCR awareness may still be helpful when the LCR impacts a gene that is particularly consistent with the diagnostic indication for the patient. We did not observe such cases in the examined NDD cohort. The lack of direct evidence of LCR utility may be due to the extreme genetic heterogeneity of NDDs, which makes it unlikely that an LCR would be matched to a disease-causing variant in a small-sized cohort. Nonetheless, this limitation does not diminish the potential impact of LCR analysis. Moreover, concepts and analyses from this work readily apply to other RGDs. Beyond the risk of uncalling, LCRs can negatively affect allele and genotype quality, leading to mistaken filtering of the variants in downstream analysis [22,23], further contributing to uncertainty in data processing in ways so subtle that they do not appear in global sequencing coverage metrics. Although we used a single ES enrichment method, the capture kit we studied (Twist v1.3) delivers superior coverage uniformity and performance compared to other commercial kits [1]. We used different bioinformatic pipelines for ES and GS, leaving the possibility that pipeline-specific factors affect our conclusions. However, the pipelines we used are both widely adopted in clinical workflows and therefore represent real-world scenarios that are nonetheless useful for comparison. Moreover, potential pipeline specificities are limited to the alignment and mapping process, for which the two algorithms used are known to ensure comparable high performances. The definition of LCRs relied on mean DoC < 20 x, while z-scores < −1.96 were subsequently used to identify, among LCRs, those showing consistent undercoverage within a batch and to compare coverage patterns across batches. While the 20 x threshold is consistent with generally adopted recommendations for variant detection in NGS data, it is still arbitrary; similarly, the z-score threshold may yield different results with alternative statistical cutoffs. Consequently, LCR patterns and their frequency may shift with different coverage or z-score thresholds. To address this concern, we evaluated alternative depth cut-offs (10 x and 30 x) for the ES-GS comparison and compared several z-score thresholds (z ≤ −1.64, −1.96, and −2.58) for the definition of systematic LCRs. While these choices affected the absolute number of intervals classified as LCRs, the qualitative patterns, including the limited overlap between ES and GS-specific LCRs and the presence of batch-dependent LCR profiles, remained consistent (Figures S2 and S8, Tables S1–S4). These findings suggest that the observed differences between the two sequencing approaches, as well as the batch-dependent patterns seen within ES, are not driven by the specific threshold definition but remain consistent across the alternative coverage and z-score cut-offs evaluated in this study. As our analyses were mainly centered on ES and used GS only for comparison, we have not extended the batch analysis to different GS runs. However, batch effects can also be reasonably present in GS data, and this aspect warrants further thorough analysis in future work, including the analysis of non-coding regions. Finally, prospective evaluation of the clinical utility of LCR reporting would reinforce the evidence for adoption in routine diagnostics.

In summary, LCRs are likely to be a relatively frequent feature in acceptably high mean DoC experiments with most target bases having >20 x coverage. Our findings underscore the need for systematic LCR assessment, warning about regions of the exome or the genome where sequencing failures may obscure disease-causing variants, especially in heterogeneous genetic diseases such as NDDs, where the large gene target in any exome or genome sequenced is particularly susceptible to LCR occurrence.

## Figures and Tables

**Table 1:** Summary of samples included in the study. The study included 43 Twist ES samples distributed across four sequencing batches. A subset of 14 of these samples was additionally sequenced by GS, enabling direct comparison of low-coverage regions between ES and GS.

Dataset	Kit/Methods	Read Length	Batch Mean DoC	N Samples	Purpose
Twist ES-Batch 1	Twist v1.3	150 × 2	229 x	10	Batch-effect analysis
Twist ES-Batch 2	Twist v1.3	150 × 2	117 x	12	Batch-effect analysis
Twist ES-Batch 3	Twist v1.3	150 × 2	117 x	11	Batch-effect analysis
Twist ES-Batch 4	Twist v1.3	150 × 2	126 x	10	Batch-effect analysis
Twist ES-GS subset	Twist v1.3	150 × 2	225 x	14	ES/GS LCRs comparison
GS-ES subset	na	150 × 2	64.4 x	14	ES/GS LCRs comparison

**Table 2:** Intra-batch consistency of LCRs. For each batch, intervals were classified as consistent when all samples agreed on their status (LCR or non-LCR).

Batch	% Consistence	Consistence	Inconsistent	tot
batch 1	98%	11,684	227	11,911
batch 2	81.5%	9705	2206	11,911
Batch 3	80%	9537	2374	11,911
Batch 4	84.5%	10,068	1834	11,911

## Data Availability

The original contributions of this study are included in the article and its Supplementary Material. Detailed information about the commands used to generate the intermediate datasets and the R scripts used for downstream analyses is available in the GitHub repository: https://github.com/emanuela04/coveragES-coveraGeS_analysis, accessed on 1 January 2026. Further inquiries can be directed to the corresponding author.

## Supplementary Materials

The following supporting information can be downloaded at: https://www.mdpi.com/article/10.3390/genes17030269/s1, Figure S1. Analytical workflow for identification and clinical assessment of LCRs, from ES/GS CRAM files to coverage calculation on GENCODE CDS intervals, LCRs classification, functional annotation with unCOVERApp, and manual review of OMIM genes against patient HPO terms. Figure S2. Per-sample Jaccard similarity coefficients between ES and GS low-coverage regions are shown for three coverage thresholds (10 x, 20 x, and 30 x). Figure S3. Per-sample counts of ES-only, GS-only, and shared LCRs in paired ES–GS samples, illustrating heterogeneous and sequencing methods-specific low-coverage regions across individuals. Figure S4. Gene-level LCR burden normalized by CDS length (LCRs per kb), showing the relative contribution of ES-only, GS-only, and shared LCRs across clinically relevant genes with the highest normalized low-coverage rates. Figure S5. Example visualization of PIGN in uncoverappLib, illustrating DoC relative to the 20 x threshold and transcript structure across the gene. Figure S6. Visualization of consistent regions and inconsistent regions across all 4 batches. Figure S7. PCA of binary LCR profiles across exome batches, showing that samples cluster largely by batch, consistent with batch-dependent low-coverage signatures. Figure S8. Intra-batch LCR consistency across alternative z-score thresholds, indicating that the relative differences in consistency among ES batches remain stable, supporting the robustness of batch-dependent patterns. Table S1. Summary statistics of ES-GS low-coverage overlap across coverage thresholds: the table reports the number of samples, median, and interquartile range of Jaccard similarity coefficients, and the average number of ES-only, GS-only, and shared LCRs per individual. Table S2. Per-sample summary of LCRs in paired ES–GS data, including total and SysNDD-overlapping LCR counts, median LCR depth, and numbers of affected genes overall, in OMIM and in SysNDD. Table S3. Gene-level LCR summary reporting ES-only, GS-only, and shared LCR counts, CDS length, GC content, and length-normalized LCR rates (per kb of CDS). Table S4. Intra-batch LCR consistency for ES data across different z-score cut-offs, reporting consistent and inconsistent intervals and the resulting percentage of concordant intervals in each batch.

## Abbreviations

The following abbreviations are used in this manuscript:

ES	Exome sequencing
GS	Genome sequencing
LCRs	Low-coverage regions
CDS	Coding DNA sequence
