# Supplementary material for: Distribution of Sequencing Coverage Gaps in Exomes and Genomes: Potential Implications for Diagnostic Accuracy in Neurodevelopmental Disorder Genes
Source: Genes (Basel). 2026 Feb 26;17(3):269. doi: 10.3390/genes17030269 (PMC13025359; doi:10.3390/genes17030269)
Supplement: Supplementary file 1 [file genes-17-00269-s001.zip › Caption_supplementary_figures.pdf]

Figure S1 Analytical workflow for identification and clinical assessment of LCRs. CRAM files from ES/GS are used to compute coverage on GENCODE CDS intervals, and intervals with Doc <20x are classified as LCRs. LCRs overlapping OMIM genes are then annotated with ClinVar clinical significance and OMIM disease entries through *uncoverappLib*, and candidate genes are manually reviewed against patient HPO terms to evaluate phenotype consistency.

Figure S2. ES-GS low-coverage overlap to coverage threshold.
Per-sample Jaccard similarity coefficients between ES and GS low-coverage regions are shown for three coverage thresholds (10x, 20x, and 30x). More permissive thresholds (10x) capture a limited set of severely undercovered intervals, some of which are shared between platforms, whereas more stringent thresholds (30x) include a larger number of marginally covered regions and amplify platform-specific differences in coverage performance. As a result, ES-GS overlap remains low with increasing threshold stringency, demonstrating that the observed discordance is robust to cutoff choice.

Figure S3 Sample-level distribution of ES-only, GS-only and shared LCRs. Barplot showing, for each of the 140 individuals sequenced by both exome sequencing (ES) and genome sequencing (GS), the number of LCRs detected exclusively in ES, exclusively in GS or in both methods. The heterogeneous balance of ES-only, GS-only and shared LCRs across samples illustrates that ES and GS capture largely distinct sets of low-coverage regions within the same individuals, without a consistent trend favoring one method over the other.

Figure S4. Gene-level LCRs burden normalized by coding sequence length across clinically relevant genes.
Normalized LCR burden (LCRs per kb of coding sequence) for the top-ranking genes associated with neurodevelopmental disorders (NDD; top 25, upper panel) and OMIM phenotype-associated genes not classified as NDD (OMIM-only; top 25, lower panel).
Genes are ranked by total normalized LCR burden across sequencing categories. Bars represent the contribution of ES-only, GS-only, and shared LCRs. Gene names are highlighted in red for NDD-associated genes and in blue for OMIM-only genes.

Figure S5. **Visualization of LCRs in uncoverppLib for PIGN gene.**

Assessment of the PIGN gene in a sample from the cohort included in this study, generated using *uncoverappLib*. The upper panel shows a chromosome ideogram indicating the chromosomal location of the gene. The sequencing depth track displays target intervals along the x-axis and the corresponding coverage values on the y-axis. Different colors indicate regions with coverage above or below the threshold of 20x which is user-defined and represented by the horizontal line. The lower panel shows the gene annotation track from ucsc, including all annotated transcripts for the gene.

Figure S6. Number of consistent and inconsistent low-coverage regions per batch. Consistent regions show the same low-coverage status across all samples within a batch, whereas inconsistent regions display intra-batch variability

Figure S7. Principal Component Analysis (PCA) of low-coverage profiles across batches. Each point represents a sample, colored by sequencing batch. Batch 1 forms a compact, distinct cluster, whereas Batches 2-4 show partially overlapping but still discernible structures. Although the separation is not absolute, samples tend to group according to batch, indicating that LCRs profiles retain a detectable batch-dependent signature rather than reflecting purely random variation.

Figure S8. Intra-batch LCR consistency to the z-score cutoff.

For each Twist-ES sequencing batch, genomic intervals were classified as low-coverage based on batch-normalized z-scores using three commonly adopted thresholds ($z \leq -1.64$, -1.96, and -2.58, corresponding to one-sided 90%, 95%, and 99% confidence levels, respectively). Bars indicate the percentage of genomic intervals for which all samples within a batch concordantly agree on their low-coverage status (LCR or non-LCR). Across all cutoffs, Batch 1 consistently shows near-complete concordance, whereas the other batches display lower but stable levels of intra-batch consistency. Importantly, while increasing stringency reduces the total number of intervals classified as low-coverage, the relative differences among batches remain preserved, demonstrating that batch-dependent patterns of low coverage are robust to variation in the z-score threshold.
