# Supplementary material for: Distribution of Sequencing Coverage Gaps in Exomes and Genomes: Potential Implications for Diagnostic Accuracy in Neurodevelopmental Disorder Genes
Source: Genes (Basel). 2026 Feb 26;17(3):269. doi: 10.3390/genes17030269 (PMC13025359; doi:10.3390/genes17030269)
Supplement: Supplementary file 1 [file genes-17-00269-s001.zip › caption_Table_supplementary.pdf]

TabS1. **Summary statistics of ES-GS low-coverage overlap across coverage thresholds**.For each threshold, the table reports the number of samples, median and interquartile range of Jaccard similarity coefficients, and the average number of ES-only, GS-only, and shared LCRs per individual.

TabS2. **Summary of low-coverage regions (LCRs) identified in the 14 paired-sample.** The table is reported across two pages: the first page includes results for the exome sequencing (ES) dataset, and the second page includes results for the genome sequencing (GS) dataset. For each sample, the table reports the total number of LCRs (n_LCR_total), the number of LCRs overlapping SysNDD-associated genes (n_LCR_SysNDD), the total number of base pairs affected by low coverage (total_LCR_bp), the total number of base pairs affected within SysNDD-associated genes (total_LCR_SysNDD_bp), the median depth of coverage across LCRs (median_LCR_depth), and the number of unique genes affected by at least one LCR overall, in OMIM genes, and in SysNDD genes (n_genes_LCR, n_genes_LCR_OMIM, n_genes_LCR_SysNDD).

TabS3. **Gene-level summary of LCRs.** This table reports gene-level counts of LCRs identified in the dataset, stratified by category (ES-only, GS-only, and Shared). For each gene, raw LCR counts are provided together with coding sequence length (CDS, in base pairs), GC content, and LCR rates normalized per kilobase of CDS. Normalized values enable comparison of LCR burden across genes while accounting for differences in gene length and sequence composition.

TabS4. I**ntra-batch low-coverage region (LCR) consistency to the z-score cutoff.** For each Twist-ES sequencing batch, genomic intervals were classified as low coverage using three alternative batch-normalized z-score thresholds ($z \leq -1.64$, -1.96, and -2.58). For each cutoff, the table reports the number of intervals for which all samples within a batch showed concordant coverage status (consistent), the number of intervals with discordant behavior across samples (inconsistent), the total number of intervals evaluated, and the resulting percentage of consistent intervals.

Although increasing stringency reduces the number of intervals classified as low coverage, the relative differences in intra-batch consistency among batches remain stable across thresholds, supporting the robustness of batch-dependent coverage patterns.
