# Supplementary figures and images for: Distribution of Sequencing Coverage Gaps in Exomes and Genomes: Potential Implications for Diagnostic Accuracy in Neurodevelopmental Disorder Genes
Source: Genes (Basel). 2026 Feb 26;17(3):269. doi: 10.3390/genes17030269 (PMC13025359; doi:10.3390/genes17030269)

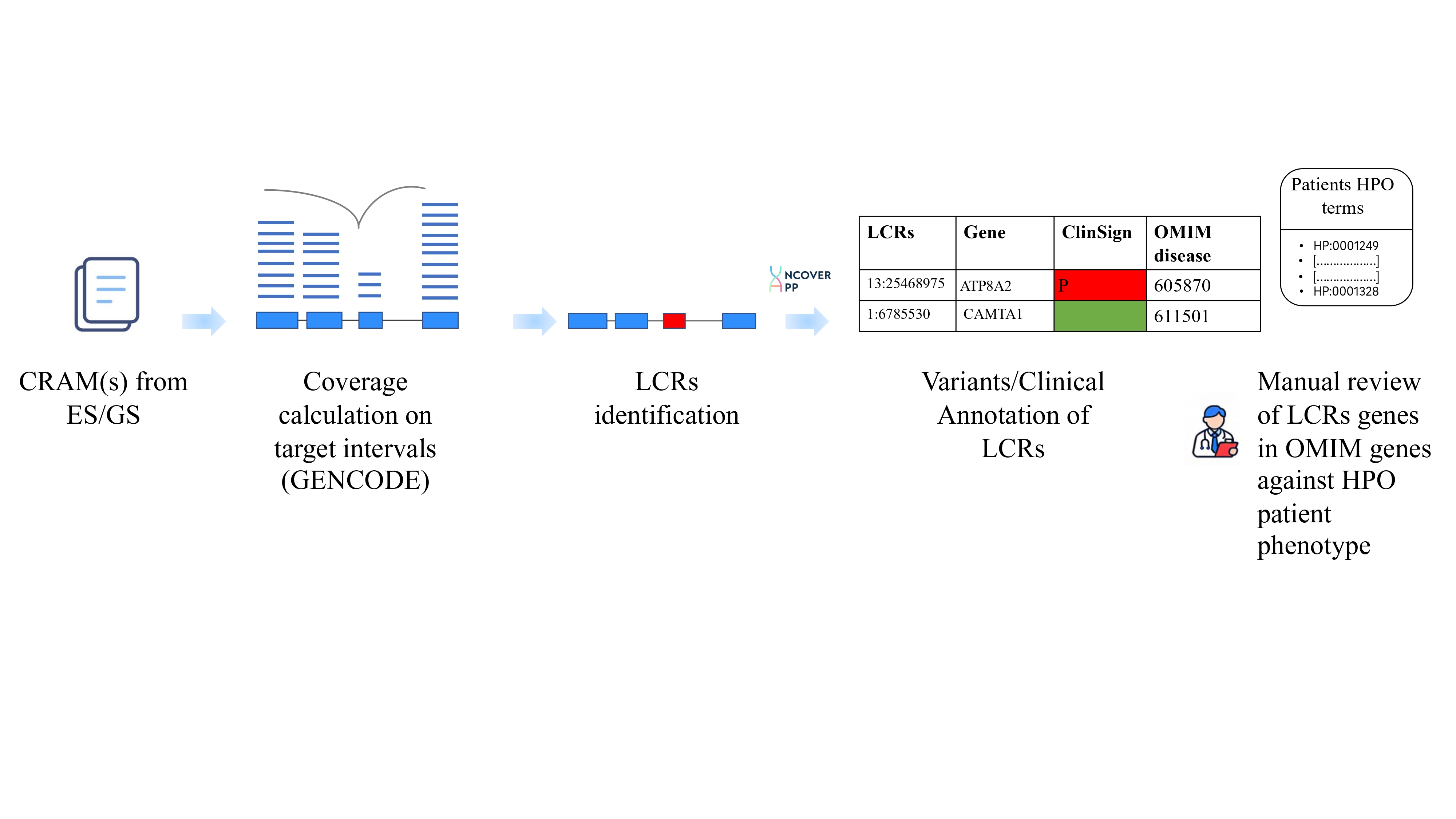

Supplement: Supplementary file 1 [file genes-17-00269-s001.zip › FigureS1.jpg]

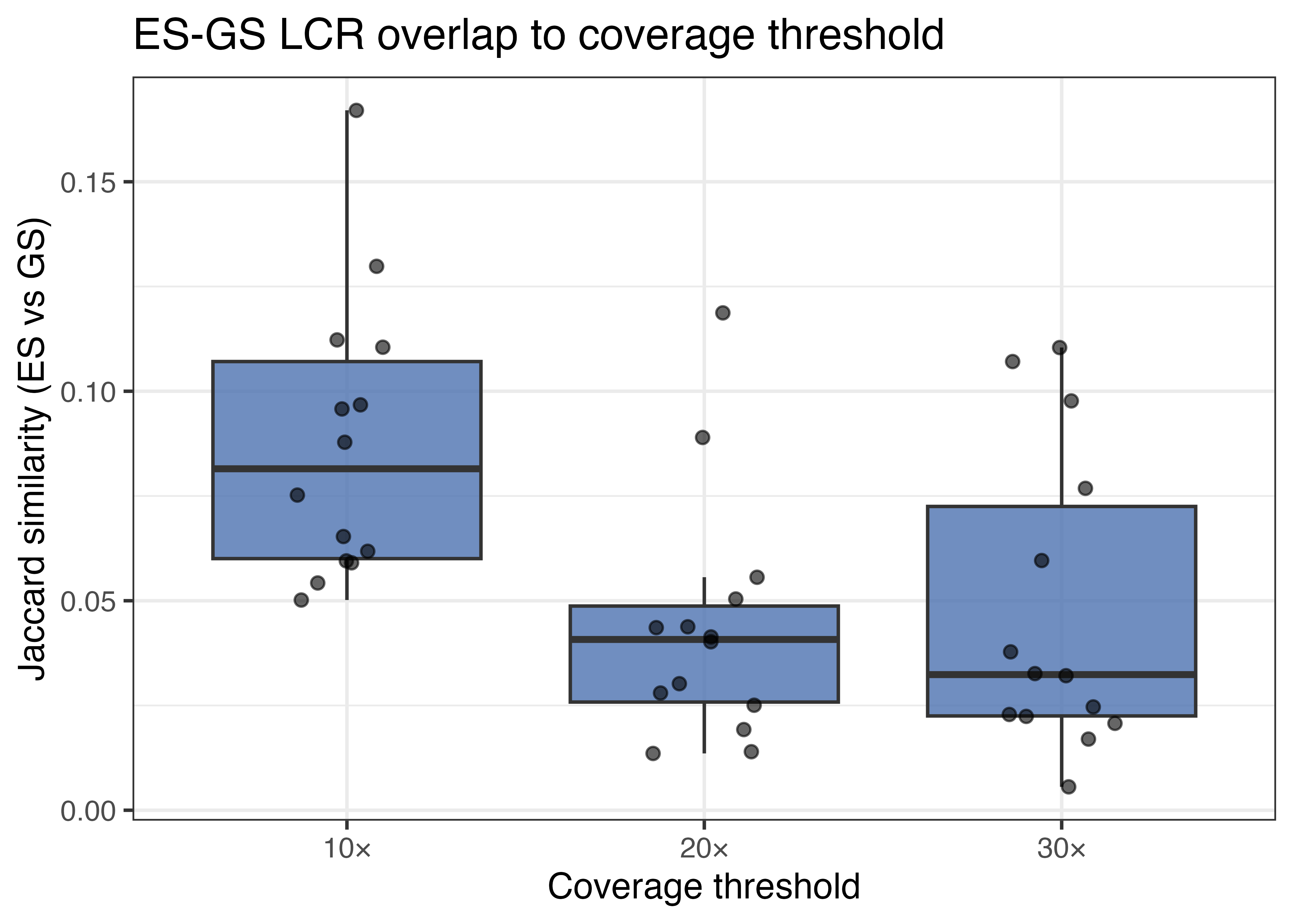

Supplement: Supplementary file 1 [file genes-17-00269-s001.zip › FigureS2.png]

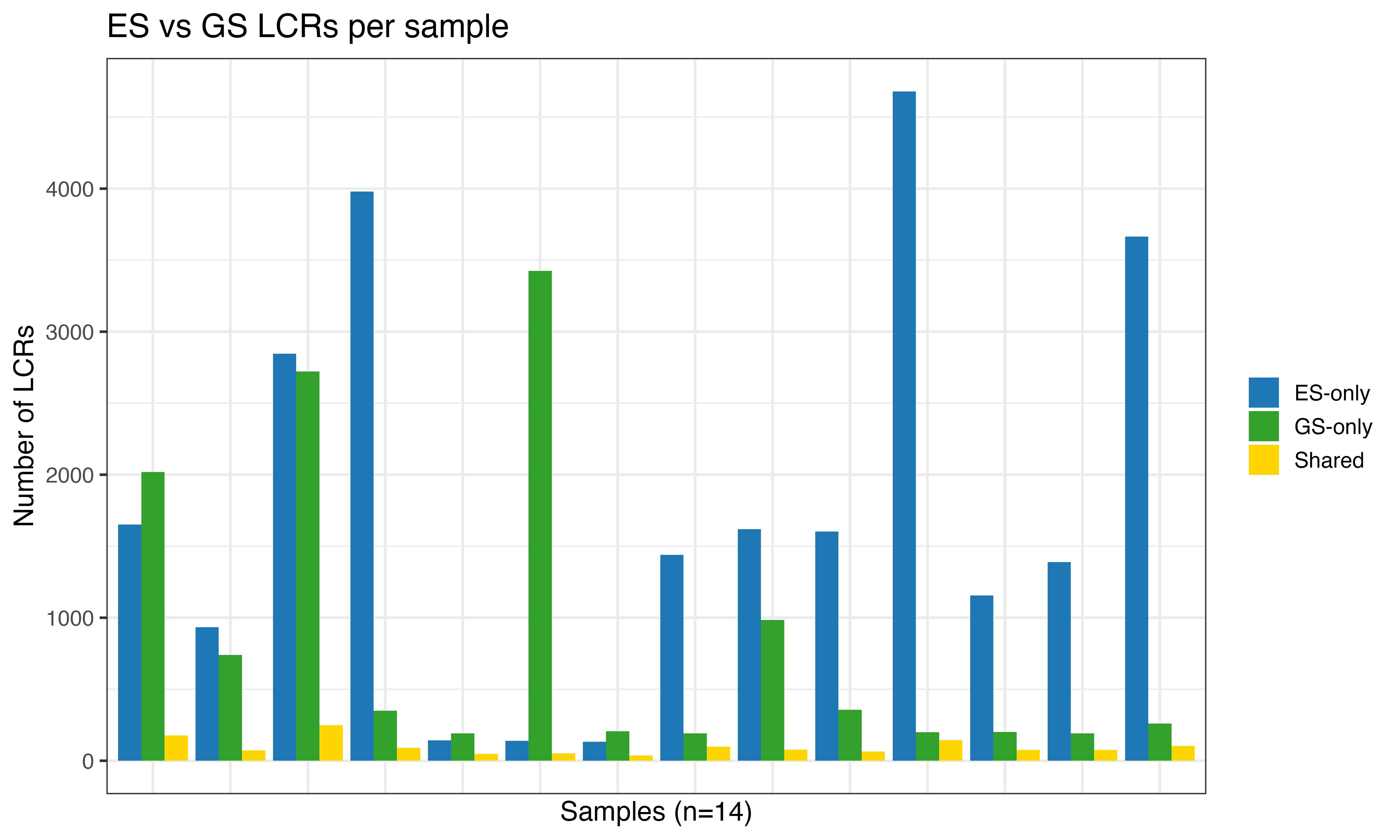

Supplement: Supplementary file 1 [file genes-17-00269-s001.zip › FigureS3.png]

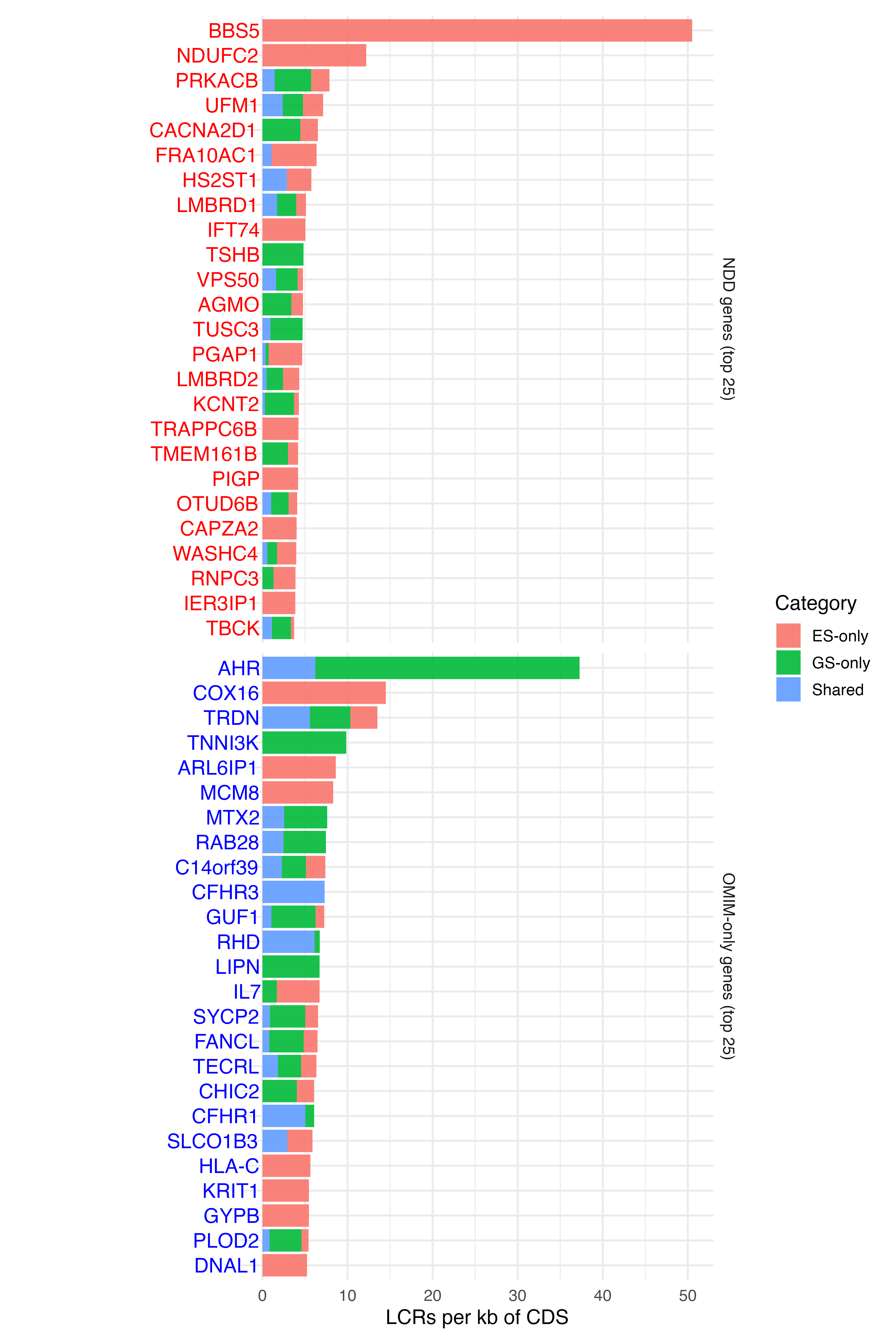

Supplement: Supplementary file 1 [file genes-17-00269-s001.zip › FigureS4.png]

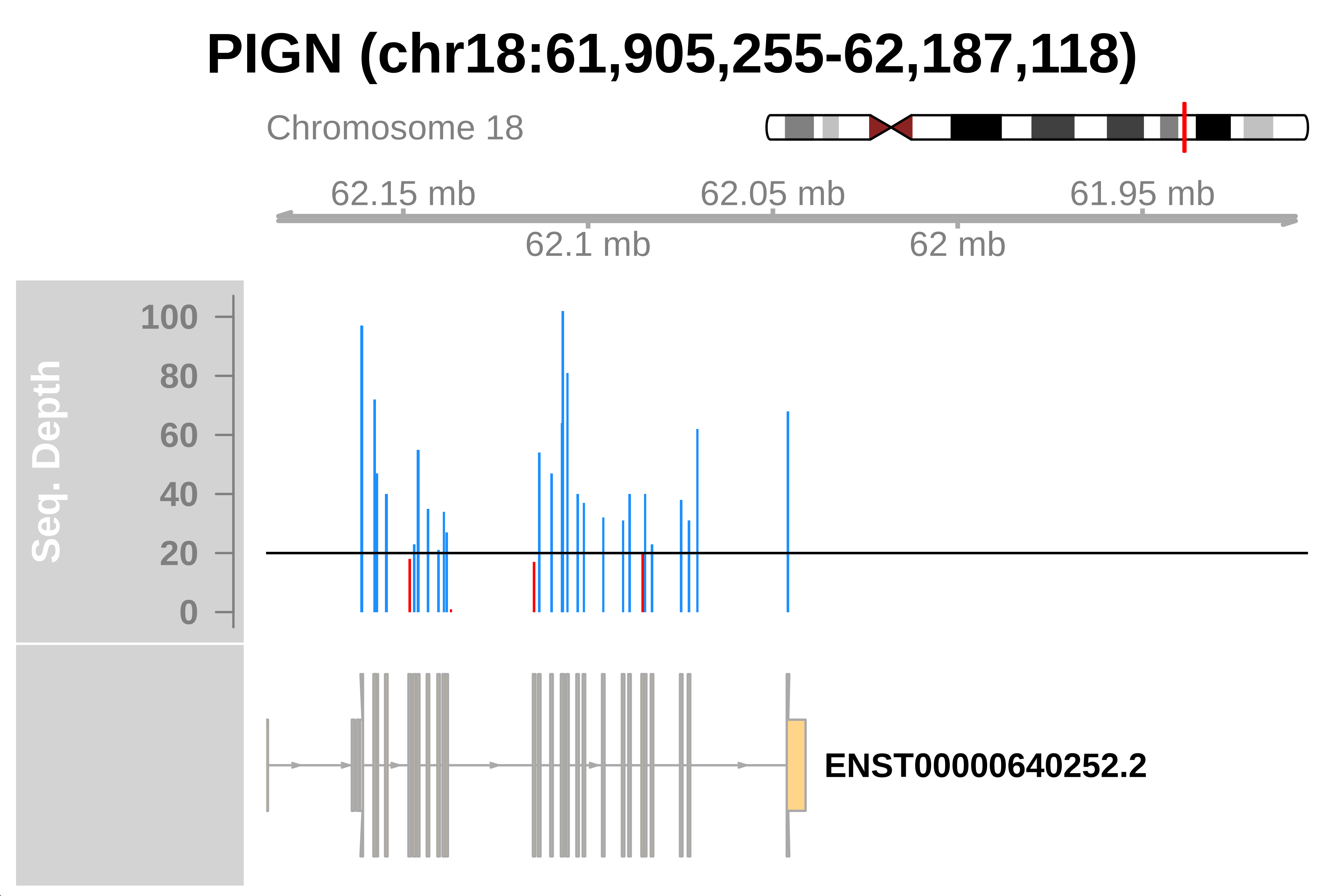

Supplement: Supplementary file 1 [file genes-17-00269-s001.zip › FigureS5.png]

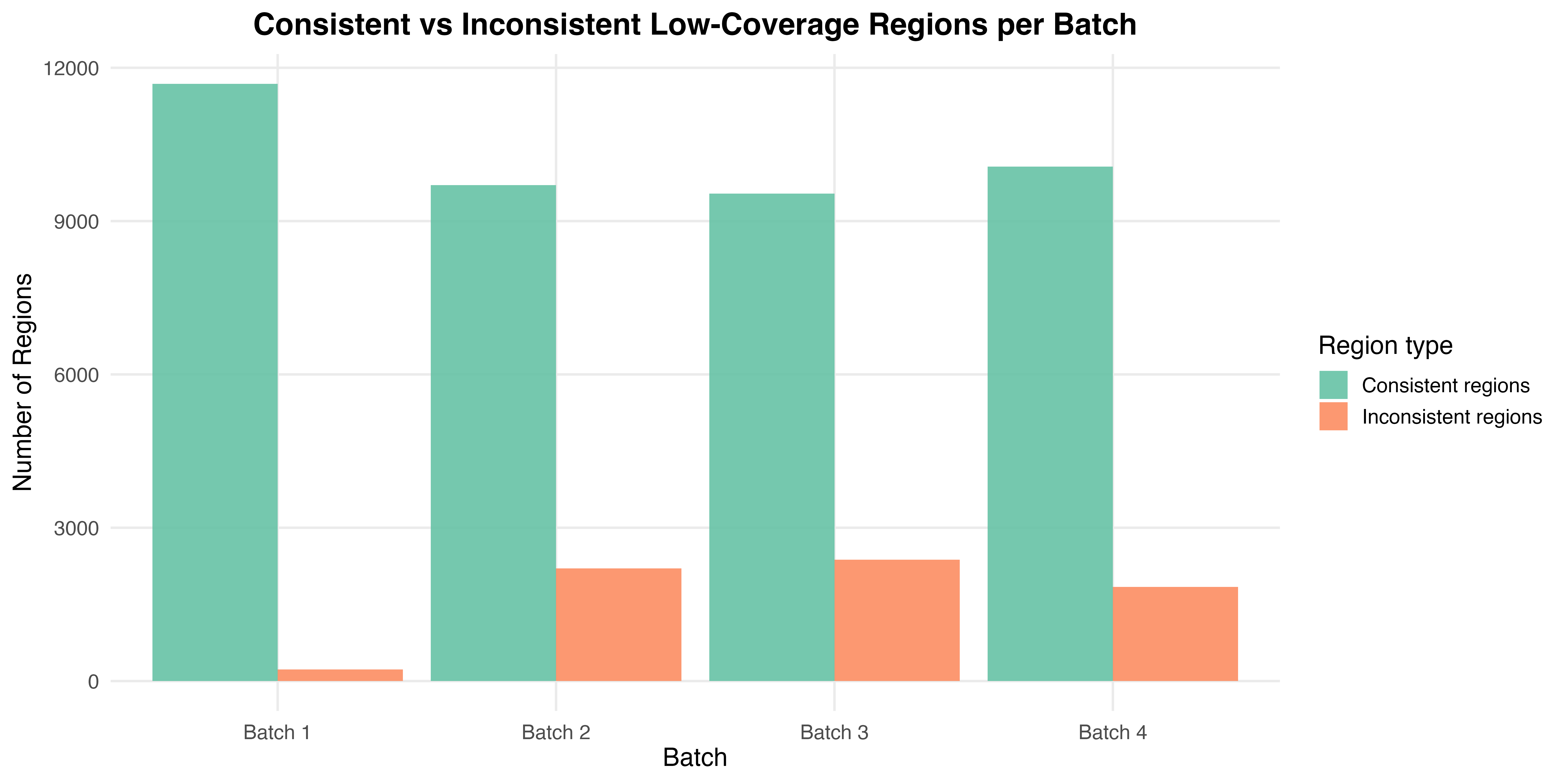

Supplement: Supplementary file 1 [file genes-17-00269-s001.zip › FigureS6.png]

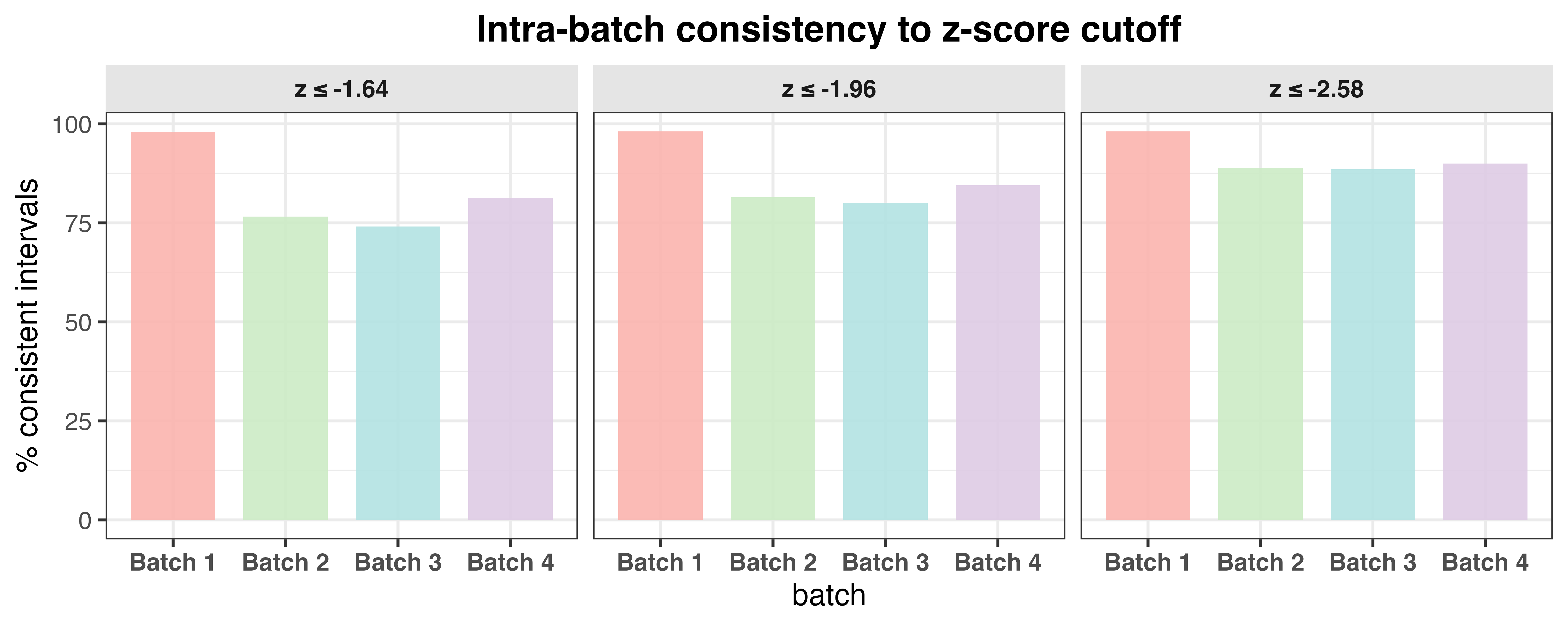

Supplement: Supplementary file 1 [file genes-17-00269-s001.zip › FigureS7.png]
